# Maturation of the QT Variability Index is Impaired in Preterm Infants

**DOI:** 10.1007/s00246-018-1839-2

**Published:** 2018-03-12

**Authors:** Arisa Kojima, Tadayoshi Hata, Tsuneaki Sadanaga, Yuri Mizutani, Hidetoshi Uchida, Yuri Kawai, Masahiko Manabe, Masayuki Fujino, Yoshihiko Eryu, Hiroko Boda, Masafumi Miyata, Tetsushi Yoshikawa

**Affiliations:** 1 Department of Pediatrics, School of Medicine, Fujita Health University, Toyoake, Japan; 2 Graduate School of Health Sciences, Fujita Health University, 1-98, Dengakugakubo, Kutsukake-cho, Toyoake, Japan; 3 Seigato Hospital, Kumamoto, Japan

**Keywords:** QT variability index, Cardiac autonomic nervous system, Preterm infants, Gestational age

## Abstract

Reduced heart rate (HR) variability in preterm infants compared with full-term infants suggests that autonomic cardiac control is developmentally delayed. However, the association between developmental changes in myocardial repolarization and gestational age remains unknown. This study investigated the association between the myocardial repolarization lability index, namely the QT variability index (QTVI) = log_10_ [(QTv/QTm^2^)/(HRv/HRm^2^)], and the perinatal profile of healthy 1-month-old infants. We included 209 infants (143 boys and 87 girls; mean gestational weeks at birth, 38.6 ± 1.7) who were born in university hospitals between 2014 and 2015 without apparent cardiac disease. We compared the ECG variability indices in 28 infants born before 37 gestational weeks (mean gestational weeks at birth, 35.6 ± 1.1 as preterm) and 181 infants born at the average number of gestational weeks (mean gestational weeks at birth, 38.8 ± 1.1 as controls). There was a negative correlation between the QTVI and gestational weeks (*r* = − 0.460, *p* = 0.035). QTVI values in preterm infants were larger than those in the controls (0.01 ± 0.50 vs. −0.26 ± 0.48, *p* = 0.023). In conclusion, the QTVI is negatively correlated with gestational age. The QTVI can serve as an index of the maturity of the cardiac autonomic nervous system and myocardial depolarization.

## Introduction

Japan is currently the country with the lowest neonatal mortality rate in the world [1, 2]. However, in the fiscal year of 2015, 96 infants in Japan died because of sudden infant death syndrome (SIDS), for which there were no warning signs or past history. Therefore, SIDS is the third highest cause of death during infancy [3]. Although the pathophysiology of SIDS is still under investigation, dysfunction of the autonomic nervous system, including the respiratory and cardiovascular systems, may be involved [4, 5].

Heart rate variability (HRV) reflects the complex interplay of sympathetic and parasympathetic innervation of the heart [6, 7]. HRV before birth in infants who subsequently died of SIDS was reported to have low frequency power [8, 9]. This finding suggests that preterm infants with decreased HRV are at a high risk for SIDS.

Action potential duration, as reflected by the QT interval, is modulated by a previous cardiac cycle, and the QT interval varies according to the RR interval [10, 11]. However, few studies have investigated the myocardial repolarization process in infants, which enables estimation of the effect of the growth of autonomic nerves to the ventricular myocardium [12]. The QT variability index (QTVI), which is used as an index of the variable ratio of the RR and QT intervals, is easily calculated from a single lead using a well-defined algorithm. Therefore, the QTVI is practically applicable if the ECG record has a stable baseline [13].

We have previously reported developmental changes in the QTVI using body surface ECG in subjects from infants to school age [12]. In this study, we aimed to clarify our hypothesis that QTVI values for 1-month-old infants are affected by gestational age, which reflects embryonal development.

## Subjects and Methods

We screened 230 infants who were born at Fujita Health University Hospital between October 2014 and November 2015 and underwent 1-month outpatient examinations. In this study, 20 infants (1 preterm infant and 19 controls) were excluded because terminal T portion and P wave were overlapped due to extremely high heart rates (> 180 beats/min) and the end of T wave could not be accurately measured. One infant (control infants) with patent ductus arteriosus was also excluded. The remaining 209 subjects were enrolled. The subjects’ mean age was 32.9 ± 4.0 days, and their mean gestational age (GA) was 38.4 ± 1.6 weeks. The parents or guardians provided written informed consent.

ECGs were recorded using a CM5 lead and a Biopac biological polygraph recording device model MP-150 (Biopac Systems Inc., CA, USA). The recordings were performed between 2 and 3 pm in the afternoon before breastfeeding in the supine position while the subjects were quiet and awake. The RR intervals for the recordings of 60 heartbeats with stable baselines were automatically analyzed using the analytical software AcqKnowledge version 3.9 (Biopac Systems Inc.). Primary differentiation processing and absolute value processing were then performed using ECG waveforms. T-wave endpoints were determined, and RR and QT intervals for the same heart rates (HRs) were measured. The means and variances were calculated for the target HRs for each time point. We then calculated QTVI (log_10_ [(QTv/QTm^2^)/(HRv/HRm^2^)], Berger et al. [13, 14]) using mean HR (HRm), HR variance (HRv), mean QT interval (QTm), and QT variance (QTv). Corrected QT intervals were calculated using Bazett’s and Fridericia’s correction formulas.

Based on the recommendations of the American College of Obstetricians and Gynecologists [15], we defined a preterm infant as an infant born at a GA of < 37 weeks. The remaining subjects served as the control group.

### Statistical Analysis

The statistical software package JMP (version 8; SAS Institute, Cary, NC, USA) was used for the analyses. Data are presented as the mean ± standard deviation. The event frequencies were compared using the *χ*^2^ test. Comparisons between the two groups of data were made using the unpaired Student’s *t* test or Wilcoxon ranked test as appropriate. Correlations between the variability indices and the number of gestational weeks were evaluated using linear regression analysis. A *p* level of < 0.05 was considered statistically significant.

## Results

### Participants’ Characteristics

Table 1 shows the characteristics of the preterm infants and controls. Body weight was lighter and height was lower at birth and at 1-month examinations in preterm infants compared with those in control group.

**Table 1:** Characteristics of the subjects

	< 37 weeks	≥ 37 weeks	*p* value
Number	28	181	
Male/female	16/12	109/72	0.831
Gestational age (week)	35.6 ± 1.1	38.8 ± 1.1*	< 0.0001
Birth weight (g)	2330.3 ± 294.7	3008.1 ± 374.3*	< 0.0001
Birth height (cm)	45.4 ± 2.4	48.6 ± 2.0*	< 0.0001
1 month weight	3181.8 ± 474.8	4010.3 ± 544.0*	< 0.0001
1 month height	49.2 ± 2.5	52.5 ± 2.1*	< 0.0001

Each value is expressed as the mean ± SD

*p < 0.05 with the Student *t* test

### ECG Parameters in Preterm and Control Infants

There were no significant differences in any of the ECG parameters between the two groups (Table 2).

**Table 2:** Comparison of ECG parameters

	< 37 weeks (*n* = 28)	≥ 37 weeks (*n* = 181)	*p* value
HR (bpm)	167.8 ± 16.5	163.1 ± 18.2	0.197
RR (ms)	361.3 ± 36.7	373.1 ± 43.4	0.172
QRS (ms)	63.7 ± 5.7	64.6 ± 4.5	0.301
QT (ms)	246.1 ± 18.6	250.0 ± 20.0	0.339
QTcB (ms)	409.6 ± 13.9	409.7 ± 17.7	0.984
QTcF (ms)	345.5 ± 15.8	347.3 ± 17.9	0.615

*QTcB* corrected QT intervals by Bazett’s formula, *QTcF* corrected QT intervals by Fridericia’s formula. Each value is expressed as the mean ± SD. There was no significance with the Student *t* test

### Correlations Between the QTVI and Perinatal Profiles

Table 3 shows linear regression analysis between the QTVI and GA, birth weight, and the Apgar score (1 and 5 min). A significant correlation (*r* = − 0.46, *p* = 0.035) between the QTVI and GA (weeks) was observed (Table 3).

**Table 3:** Correlations between the QTVI and perinatal profiles

	*r*	*p*
GA	− 0.460	0.035
BW	0.066	0.341
Apgar (1 min)	0.035	0.621
Apgar (5 min)	0.058	0.399

Each value is expressed as the mean ± SD. There was no significance with the linear regression analysis

*GA* gestational age, *BW* birth weight

### Comparison of Variability Indices

Table 4 shows comparison of several variability indices between preterm infants and controls. There were no significant differences in HRm, QTm, QTv, and normalized QT variability between the two groups. However, HRv and normalized HR variability were significantly lower in preterm infants than in controls (*p* = 0.038, 0.026, respectively). The QTVI was significantly higher in preterm infants than in controls (*p* = 0.023).

**Table 4:** Comparison of variability indices

	< 37 weeks (*n* = 28)	37 weeks ≤ (*n* = 181)	*p* value
HR mean			
Mean ± SD	167.8 ± 16.5	163.1 ± 18.2	0.193
Median	171.2	164.0	
HR Var			
Mean ± SD	22.5 ± 25.0	30.9 ± 29.2*	0.038
Median	9.9	20.4	
QT mean			
Mean ± SD	246.1 ± 18.6	249.9 ± 20.0	0.338
Median	242.8	248.8	
QT Var			
Mean ± SD	31.7 ± 16.9	30.7 ± 19.8	0.792
Median	31.3	24.3	
HRVN (10^3^)			
Mean ± SD	0.84 ± 0.93	125 ± 1.28*	0.026
Median	0.37	0.79	
QTVN (10^3^)			
Mean ± SD	0.53 ± 0.30	0.50 ± 0.34	0.423
Median	0.49	0.42	
QTVI			
Mean ± SD	0.01 ± 0.50	− 0.26 ± 0.48*	0.023
Median	− 0.20	− 0.29	

Each value is expressed as the mean ± SD

*HRm* mean heart rate, *HRv* variance of HR, *QTm* mean QT interval, *QTv* variance of QT intervals, *HRVN* normalized HR variability, *QTVN* normalized QT variability, *QTVI* QT interval variability index

*p < 0.05 with the Wilcoxon ranked test

## Discussion

In this study, we analyzed ECGs of 1-month-old infants and evaluated ventricular repolarization variability using the QTVI. We found that the QTVI was impaired in preterm infants and it was inversely related to GA.

We have previously reported that the QTVI might indicate postnatal autonomic nerve development and ventricular repolarization maturity [12, 16]. Our study indicates that QTVI might also reflect prenatal and/or early postnatal automatic nerve development. Preterm infants may have a maturation process of nerves that differs from that of full-term infants. The current study provided evidence that QTVI could be used as a new assessment tool for evaluating autonomic dysfunction in preterm infants.

Dysfunction of the autonomic nervous system is one of the causes of SIDS [4]. Victims of SIDS have decreased vagal nerve activity or increased sympathetic nervous system activity, as well as a long QT interval, low HRV, and high basic HR [17]. Whether the QTVI is impaired in patients with SIDS is unknown.

The QTVI is used for predicting cardiac events in adults with pathological hearts [13, 18]. In the future, we intend to investigate whether QTVI could be used to risk-stratify infants with heart disease.

### Limitations

The first limitation of this study is that all ECGs were recorded in the supine position while infants were awake and alert. Therefore, whether results were the same while subjects were asleep is unknown. Second, we did not have any information of fetal factors, placental/umbilical factors, and maternal factors, which might have affected the results. Third, we did not have any ECG data at birth. Therefore, we do not know whether QTVI is already impaired at birth in preterm infants and/or development is delayed after birth. Furthermore, because we only have ECG data of 1-month-old infants, we do not know whether there was a catch-up phenomenon (i.e., no difference in the QTVI between preterm infants and controls). This is important because preterm infants may have, at least theoretically, an increased risk for SIDS until the time of catch-up because of immaturity of autonomic function. This possibility should be investigated in a future study.

## Conclusion

The QTVI in 1-month-old infants is impaired in those who are born preterm and is inversely related to GA. The QTVI could serve as an index of the maturity of cardiac autonomic nerves and myocardial repolarization in infants.
